# Characterization of Influenza D Virus Reassortant Strain in Swine from Mixed Pig and Beef Farm, France

**DOI:** 10.3201/eid3008.240089

**Published:** 2024-08

**Authors:** Stéphane Gorin, Gautier Richard, Séverine Hervé, Eric Eveno, Yannick Blanchard, Agnès Jardin, Nicolas Rose, Gaëlle Simon

**Affiliations:** French Agency for Food, Environmental and Occupational Health & Safety, Ploufragan, France (S. Gorin, G. Richard, S. Hervé, E. Eveno, Y. Blanchard, N. Rose, G. Simon);; CEVA Santé Animale, Libourne, France (A. Jardin)

**Keywords:** Influenza D virus, influenza, viruses, pig, swine, reassortant, France, respiratory infections, zoonoses

## Abstract

Influenza D virus was isolated from pigs on a mixed pig and beef farm in France. Investigation suggested bull-to-pig transmission and spread among pigs. The swine influenza D virus recovered was a reassortant of D/660 and D/OK lineages. Reported mutations in the receptor binding site might be related to swine host adaptation.

Influenza D virus (IDV) was initially isolated in 2011 from a pig with influenza-like symptoms (*1*). IDV has been identified globally in various species, including humans, without respiratory disease association according to serologic data (*2*,*3*) and sequencing from nasal washes (*4*). Cattle are the main IDV reservoir worldwide, including in France (*5*). Sow herds in France have tested positive for IDV antibodies, but IDV has not been isolated (*6*). We describe detection of IDV in fattening pigs in France and the accompanying genetic data for the swine origin IDV strain.

## The Study

**Figure 1:**
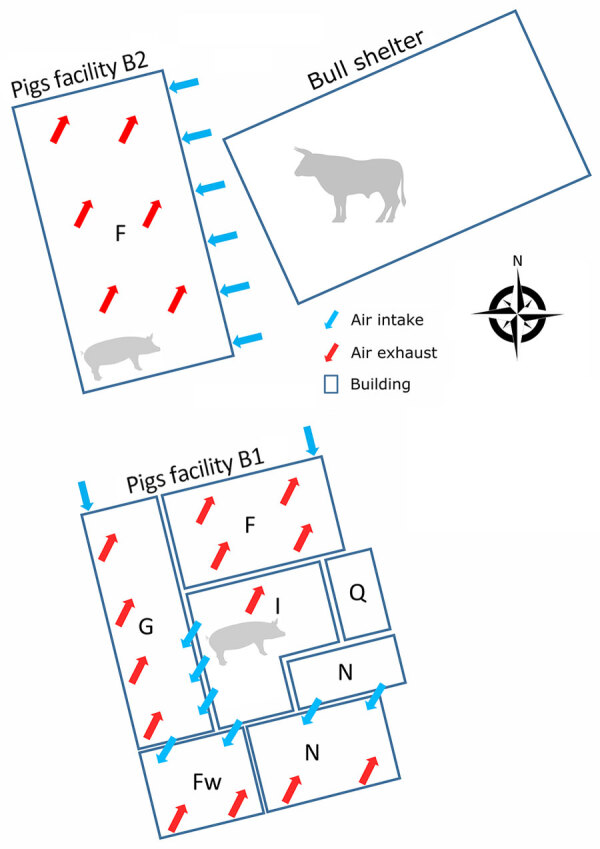
Schematic of a mixed pig and beef farm where influenza D virus was detected in pigs, France. Numbers of pigs in B1: F, n = 120 gilts; G + I, n = 160 sows and a few males; N, n = 960 (2 groups of 480 pigs, 5 and 10 weeks of age). Numbers of pigs in B2: F, n = 1,440 (3 groups of 480 pigs of 15, 20, and 25 weeks of age). F, fattening; Fw, farrowing room; G, gestation room; I, insemination room; N, nursery; Q, quarantine.

The sampled farm houses both pigs and cattle and is in the Brittany region of France. The pig herd operation is a farrow-to-finish system with 2 distinct barns: the first barn houses the sows and growing pigs in the nursery sectors and some of the fattening rooms (B1), and the other barn serves as the primary fattening barn (B2) (Figure 1). The pig herd had onset of chronic respiratory disorders that affected pigs in the nursery and fattening stages. The respiratory symptoms included sneezing and coughing.

A livestock veterinarian sampled tracheobronchial secretions from 7 fattening pigs (16 weeks of age) in October 2022. Pooled samples were submitted to the PathoSense laboratory (Ghent University, Merelbeke, Belgium) for sequencing (Oxford Nanopore Technologies, https://nanoporetech.com), which yielded results suggestive of an IDV infection. We then confirmed the presence of IDV genome in the tracheobronchial secretions by polymerase basic 1–gene qualitative reverse transcription PCR, as previously described (*1*). We successfully propagated strain D/swine/France/29-220655/2022 on swine testis cells. We confirmed viral growth by using polymerase basic 1–gene qualitative reverse transcription PCR and hemagglutination activity on chicken red blood cells (*6*).

We visited the farm in December 2022 to collect information about the layout and management. Our visit enabled us to assess the presence and spread of IDV in the pig barns. Because B2 had side air inlets close (7–10 m) to the bull shelter, we hypothesized that IDV was transmitted from the bovine reservoir to fattening pigs either through this air intake or from clothing and equipment used between both livestock sectors. We were unable to restrain the bulls for sampling, but the breeder emphasized the occurrence of respiratory signs, specifically coughing, in young bull calves. Those signs developed shortly after the arrival of the bull calves at 6 months of age. Because the calves originated from Pays de la Loire, the region with the highest serologic IDV prevalence in cattle and small ruminants in France (*7*), we considered a link between the signs and an IDV infection. We collected nasal swabs and blood samples from 30 pigs with influenza-like illness: 10 pigs from the nursery at 10 weeks of age (B1), 10 pigs from fattening at 15 weeks of age (B2), and 10 pigs from fattening at 25 weeks of age (B2). The 25-week-old pigs were from the same group as the 7 pigs sampled in October that were IDV positive. IDV genome was not detected in nasal swab samples. We tested for IDV antibodies in serum by using a hemagglutination inhibition assay and used the D/swine/France/29-220655/2022 strain as an antigen (*8*). The results indicated that the pigs exposed to IDV in October 2022 had seroconverted (Table), suggesting IDV spread within the group. In the nursery (B1), 10% of the serum was positive for IDV antibodies, indicating that IDV might also have circulated in the B1 facility among piglets or farrowing sows. The sows would have transmitted some passive immunity to piglets, and that immunity could be detected at the end of the postweaning period.

**Table:** Hemagglutination inhibition test results for the detection of influenza D virus antibodies against D/swine/France/29-220655/2022 in serum sampled from pigs housed on a mixed pig and beef farm in France, December 2022*

Growth stage, location	Age of pigs, wk	No. pigs sampled	HI titer range (mean)†	Positivity, %
Nursery, B1	10	10	<10–20	10
Fattening, B2	15	10	<10–10	0
Fattening, B2	25	10	20–160 (66)	100

*B1, nursery barn; B2, primary fattening barn; HI, hemagglutination inhibition. †Positive threshold = 20.

**Figure 2:**
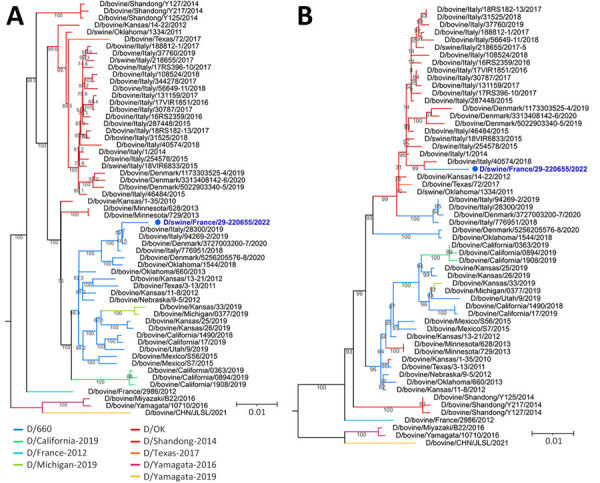
Maximum-likelihood influenza D virus phylogenetic trees displaying the isolated D/swine/France/29-220655/2022 strain (blue text) recovered from pigs at a mixed pig and beef farm in France. A) Whole-genome coding sequences phylogeny. B) Nucleoprotein coding sequences phylogeny. Numbers along branches indicate IQ-TREE2 (http://www.iqtree.org) ultra-fast bootstraps branch support. Scale bar indicates substitutions per site.

We sequenced the isolated D/swine/France/29-220655/2022 strain (Appendix) and conducted phylogenetic analysis. Our analysis revealed that the hemagglutinin-esterase fusion (HEF) sequence of D/swine/France/29-220655/2022 classified within the D/660 lineage. This lineage is closely related to contemporary bovine IDV strains in Italy but distantly related to recent bovine strains in France, which belong to the D/OK lineage (Appendix Figure). Whole-genome phylogeny also classified D/swine/France/29-220655/2022 within the D/660 lineage, again showing proximity to Italian bovine IDV strains (Figure 2, panel A). However, individual analysis of the 6 internal genomic segments revealed that 1 segment, the nucleoprotein-encoding gene, belonged to the D/OK lineage rather than D/660. This analysis indicated that D/swine/France/29-220655/2022 is a reassortant strain (Figure 2, panel B).

**Figure 3:**
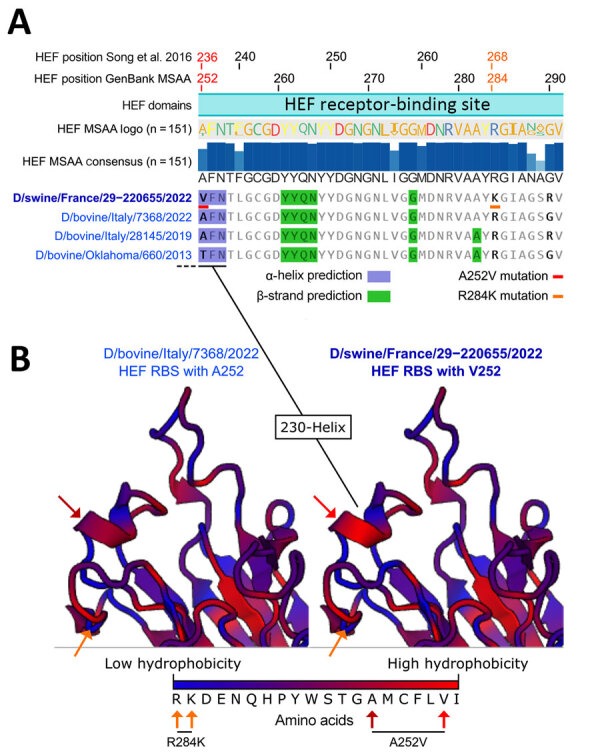
Alignments of hemagglutinin-esterase fusion protein sequences and structure prediction of the receptor-binding site of the influenza D virus strain recovered from swine in France. A) HEF sequence alignment and 2D structure prediction. From top to bottom: amino acid coordinates based on previously published research (*9*), amino acid coordinates based on GenBank MSAA and translation to protein, HEF domain based on previously published research (*9*), HEF sequence logo based on amino acid occurrence in the 151 HEF sequence alignment, HEF consensus sequence and percentage of amino occurrence in the 151 HEF sequence alignment, and representative HEF sequences from the D/660 lineage and their predicted secondary structures. Blue highlights indicate residues involved in α-helices and green highlights residues involved in β-strands. Red (A236V/A252V) and orange (R268K/R284K) underlines or arrows indicate unique mutations identified in D/swine/France/29-220655/2022. B) Predicted RBS structure of D/swine/France/29-220655/2022 compared with the closely related D/bovine/Italy/7368/2022. Blue colors on the protein 3D structures depict low hydrophobicity and red colors depict high hydrophobicity. HEF, hemagglutinin-esterase fusion; MSAA, multisequence alignment; RBS, receptor-binding site.

When aligned to the 151 publicly available IDV HEF amino acids sequences, D/swine/France/29-220655/2022 contained 2 unique mutations in the receptor binding site (RBS), A236V/A252V and R268K/R284K. We based amino-acid coordinates on previously published data (*9*) or GenBank multisequence alignment and translation to proteins (Figure 3, panel A). Those mutations are located close to the 230 helix and the 270 loop, which are essential structures forming the IDV HEF RBS open channel (*9*). Hydrophobicity is increased by the A252V mutation and can potentially change HEF RBS affinity to different receptors (Figure 3, panel B) without changing the global 3D structure of the protein.

## Conclusions

In our study, we isolated a swine IDV strain in France, confirming serologic proof of IDV circulation in pig livestock (*6*). When considering the epidemiologic investigation of the mixed pig and beef farm, we believe there was a transmission of IDV from bulls to pigs. The D/swine/France/29-220655/2022 strain displayed an HEF associated with the D/660 clade. The D/660 clade has become increasingly prevalent in Europe since 2019, after a decline in the previously dominant D/OK clade (*10*–*12*). In addition, we found that D/swine/France/29-220655/2022 was a reassortant strain that included a nucleoprotein gene from the D/OK lineage. This reassortant strain likely emerged in bovine species before transmission to swine. Reassortment could have taken place in bulls housed in the assembly center before being dispatched to fattening farms, or in a farrowing farm that delivered animals to the study farm. Either case would imply co-circulation of D/660 and D/OK clades at some point in France, similar to other European countries (*11*,*12*). Because other reassortant strains belonging to D/660 but displaying D/OK genes have been isolated from cattle in Italy (*10*), the reassortant IDV might have been imported.

Of interest, D/swine/France/29-220655/2022 HEF exhibited 2 unique mutations (A252V and R284K) located in the RBS. The HEF receptor-binding cavity is known to form an open channel between the 230 helix and 270 loop that could be responsible for IDV broad-cell tropism (*9*). By increasing HEF RBS hydrophobicity, an A252V mutation might have promoted virus binding to pig cell receptors and subsequent uptake, demonstrating an adaptation to the pig host. Single-point mutations related to hydrophobicity changes, such as threonine to isoleucine, in RBS were shown to enable adaptation to new hosts in influenza C viruses, which also could be the case for IDV HEF (*13*). The reported mutations might have contributed to the spread of the virus among pigs within the fattening unit, as shown by the serologic data.

Our case shows that IDV can be transmitted from bovines to swine and adapt to its new host through potential specific mutations enhancing intraspecies transmission. This adaptation process is similar to findings from a previous study (*14*). Replicating such a pig–calves IDV transmission experiment by using a strain such as D/swine/France/29-220655/2022 could help validate our findings. The proliferation and spread of IDV in swine could be an issue for animal health. Surveillance for IDV should be incorporated with influenza A virus surveillance, particularly in mixed pig and beef herds or in pig herds situated near bovine livestock.

Appendix Additional information about characterization of influenza D virus reassortant strain in swine from mixed pig and beef farm, France.
