# Supplementary material for: Characterization of Influenza D Virus Reassortant Strain in Swine from Mixed Pig and Beef Farm, France
Source: Emerg Infect Dis. 2024 Aug;30(8):1672–6. doi: 10.3201/eid3008.240089 (PMC11286067; doi:10.3201/eid3008.240089)
Supplement: Appendix — Additional information about characterization of influenza D virus reassortant strain in swine from mixed pig and beef farm, France. [file 24-0089-Techapp-s1.pdf]

Article DOI: https://doi.org/10.3201/eid3008.240089

EID cannot ensure accessibility for supplementary materials supplied by authors. Readers who have difficulty accessing supplementary content should contact the authors for assistance.

Characterization of Influenza D Virus Reassortant Strain in Swine from Mixed Pig and Beef Farm, France

Appendix

Methods

a. Sequencing

To assess the origins of isolated D/swine/France/29–220655/2022 strain, RNA-sequencing using the Ion Torrent Proton technology was performed both on viral RNA for in-depth study of the seven genomic segments and after hemagglutinin-esterase-fusion (HEF)-encoding gene amplification by RT-PCR using specific primers (*1*). The obtained D/swine/France/29–220655/2022 sequences we deposited in GenBank under accession numbers PP133482-PP133488.

b. Phylogenies construction

The D/swine/France/29–220655/2022 sequences were aligned to all IDV sequences available in NCBI Nucleotide database. Full-genome sequences were concatenated using ad hoc Shell scripts based on SeqKit (*2*). After alignment, non-coding and poor-quality sequences were removed. Maximum-likelihood (ML) phylogenetic trees were constructed using `iqtree2 -T AUTO–alrt 1000 -B 1000` for each segment and for the full genome alignments (*3*). The resulting ML trees were visualized, rooted at midpoint, colored and exported as SVG using the iTOL webserver (https://itol.embl.de/). The tree branches were colored based on the HEF clade classification according to Gaudino et al., 2022 (*4*), including the full-genome tree.

c. Protein 3D structure modeling

The HEF coding sequences were translated to proteins with MEGA 7.0.26 using the standard genetic code. Alignments, logo and consensus statistics were calculated and plotted using the ggmsaa R package (http://yulab-smu.top/ggmsa/). Alignments and predictions of 2D and 3D structures were performed using the Swiss-Model webserver (*5*) with the 5e64.1.A IDV HEF template (*6*).

References

1. Ducatez MF, Pelletier C, Meyer G. Influenza D virus in cattle, France, 2011-2014. Emerg Infect Dis. 2015;21:368–71. PubMed https://doi.org/10.3201/eid2102.141449

2. Shen W, Le S, Li Y, Hu F, Hu F. SeqKit: a cross-platform and ultrafast toolkit for FASTA/Q file manipulation. PLoS One. 2016;11:e0163962. PubMed https://doi.org/10.1371/journal.pone.0163962

3. Minh BQ, Schmidt HA, Chernomor O, Schrempf D, Woodhams MD, von Haeseler A, et al. IQ-TREE 2: new models and efficient methods for phylogenetic inference in the genomic era. Mol Biol Evol. 2020;37:1530–4. PubMed https://doi.org/10.1093/molbev/msaa015

4. Gaudino M, Chiapponi C, Moreno A, Zohari S, O'Donovan T, Quinless E, et al. Evolutionary and temporal dynamics of emerging influenza D virus in Europe (2009–22). Virus Evol. 2022;8:veac081. **PMID: 36533151**

5. Waterhouse A, Bertoni M, Bienert S, Studer G, Tauriello G, Gumienny R, et al. SWISS-MODEL: homology modelling of protein structures and complexes. Nucleic Acids Res. 2018;46(W1):W296–303. PubMed https://doi.org/10.1093/nar/gky427

6. Song H, Qi J, Khedri Z, Diaz S, Yu H, Chen X, et al. An open receptor-binding cavity of hemagglutinin-esterase-fusion glycoprotein from newly-identified influenza D virus: basis for its broad cell tropism. PLoS Pathog. 2016;12:e1005411. PubMed https://doi.org/10.1371/journal.ppat.1005411

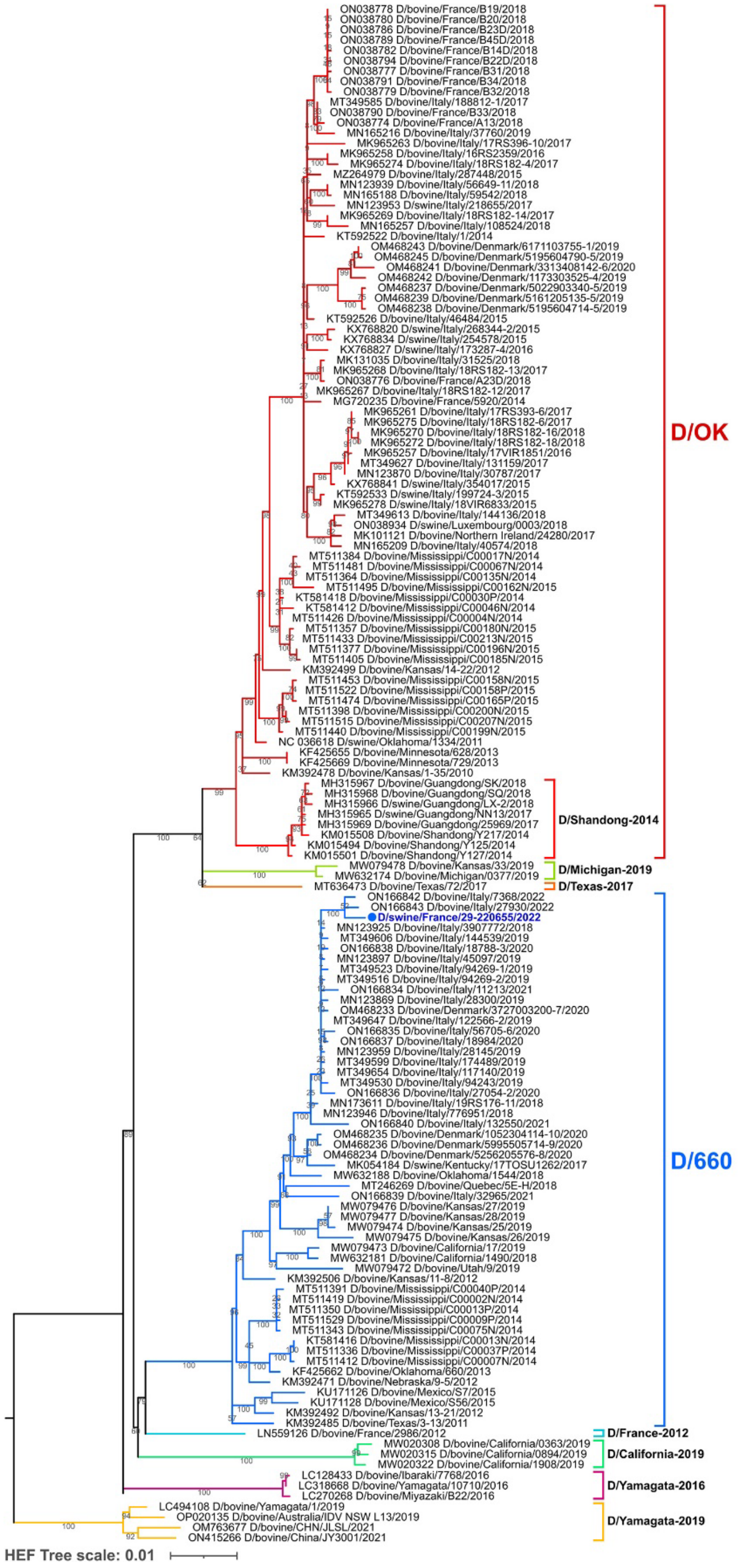

Appendix Figure. Maximum-likelihood influenza D virus phylogenetic tree created from the isolated D/swine/France/29-220655/2022 strain (blue text) recovered from pigs at a mixed pig and beef farm in France. Hemagglutinin-esterase fusion coding sequences phylogeny.
